# Inhibition of RFX6 Suppresses the Invasive Ability of Tumor Cells Through the Notch Pathway and Affects Tumor Immunity in Hepatocellular Carcinoma

**DOI:** 10.3389/fonc.2021.801222

**Published:** 2021-12-20

**Authors:** Mu Song, Mulati Kuerban, Lu Zhao, Xiaolin Peng, Youqin Xu

**Affiliations:** ^1^ Department of Surgical Oncology, The Second Affiliated Hospital, Xinjiang Medical University, Urumqi, China; ^2^ Department of Thyroid and Breast Surgery, The Seventh Affiliated Hospital, Southern Medical University, Foshan, China; ^3^ Department of Surgical Oncology, The Seventh Affiliated Hospital, Xinjiang Medical University, Urumqi, China

**Keywords:** tumorigenesis, Notch, T cells, miRNA, hepatocellular carcinoma

## Abstract

**Background:**

The DNA-binding protein RFX6 was overexpressed in hepatocellular carcinoma, and its expression level was correlated with the prognosis and immune cell infiltration in liver hepatocellular carcinoma. However, the mechanism of the abnormal expression and the biological effects of RFX6 in liver cancer remains unknown.

**Methods:**

To understand the specific expression mechanism of RFX6 in liver cancer, we performed bioinformatic prediction, CHIP-qPCR assay, co-IP, and dual-luciferase assay to assess the regulating mechanism of RFX6. In the meantime, a series of biological experiments *in vivo* and *in vitro* were conducted to analyze the biological significance of RFX6 in hepatocellular carcinoma.

**Results:**

We demonstrated that knockdown of RFX6 in liver cancer cells significantly suppressed the proliferation, migration, and invasion of cancer cells. Moreover, inhibition of RFX6 could affect the immune response of T cells. Among a number of interacting proteins, we revealed that RFX6 directly binds to DTX2, a regulator of the Notch signaling pathway by targeting NOTCH1, and helps in its transcription stability. Furthermore, we discovered that miRNA-542-3p, the expression of which was decreased in hepatocellular carcinoma, was directly involved in the negative regulation of the expression of RFX6.

**Conclusion:**

In summary, we discovered that the miRNA-542-3p–RFX6–DTX2–NOTCH1 regulatory pathway played significant roles in the tumor progression of liver hepatocellular carcinoma.

## Background

Liver cancer is the third leading cause of cancer death worldwide. Although many signaling pathways have been revealed as the key mechanisms regulating the development of liver cancer and great progress has been made in the tumor treatment, the uncontrollable progression and poor prognosis still result in high mortality. Among all types of liver cancer, hepatocellular carcinoma (HCC) is the most common type comprising 75%–85% of all cases. Hepatocellular carcinoma occurs often in men age 50 or older and is mostly caused by long-term damage of the liver, such as hepatitis B or hepatitis C virus infection, alcohol abuse, and autoimmune diseases of the liver (1). The pathogenesis study of HCC involves the genome, transcriptome, proteome and metabolism. A variety of key signaling pathways contribute to HCC tumorigenesis including the MEK/ERK (2), mitogen-activated protein kinases (MAPK) (3), mammalian target of rapamycin (mTOR) (4), JAK/STAT (5), Hedgehog (6), and TGF-β signaling pathways (7). Studies have sought to target key components in molecular and cellular pathways that are known to have aberrant signaling in HCC. While numerous targeted agents have been tested, the majority of these agents have failed to produce a survival benefit in clinical trial (8). Once HCC progresses, limited systemic treatment options are available. The resultant inflammation in combination with altered signaling pathways leads to HCC development (9).

The Notch signaling pathway is a highly conserved signaling pathway regulating a series of cell functions, including proliferation (10), apoptosis, invasion, metastasis (11), and differentiation (12). The Notch system consists of four transmembrane receptors (Notch1, 2, 3, 4) and two types of ligands: the Jagged family ligand (Jagged1, Jagged2) and the Delta family ligand (DLL-1, -3, -4) (13). Notch1, Notch3, and Notch4 are commonly overexpressed in HCC, and the activated Notch signaling pathway has also been shown to promote liver tumor formation in mouse models (14). The current study has shown that Notch1 helps promote HBV X protein-induced hepatocellular carcinoma through the Wnt/β-catenin pathway (15). The upregulation of Notch1 helps increase the carcinogenesis potential of human HCC cells (16, 17). Notch1 expression was increased in HCC and tumor metastasis (TNM) stage was significantly associated with increased Notch1 mRNA levels (18). HCC patients with TNM stage III–IV and tumor vein invasion had higher levels of Notch1 expression compared with patients with TNM stage I–II and patients without tumor vein invasion (19). Knocking out of NOTCH1 eliminated Snail1 expression and effectively inhibited the migration of cancer cells *in vitro* and in lung metastasis (17). Matrix metalloproteinase-2 (MMP-2), metalloproteinase-9 (MMP-9), and vascular endothelial growth factor (VEGF) are key factors in tumor invasion and metastasis during angiogenesis (16). The Notch signaling pathway downregulates MMP-2, MMP-9, and VEGF (12). In addition, inhibition of Notch1 has been shown to prevent HCC metastasis *in vitro* and *in vivo* (20). Overexpression of *Sox9*, a Notch pathway regulating gene, was frequently detected in HCC patients and associated with poor prognosis (14). Furthermore, CD24 promotes HCC progression *via* triggering the Notch-related EMT and modulation of the tumor microenvironment (11). Therefore, the abnormal expression of the Notch signal is closely related to the occurrence of hepatocellular carcinoma. Notch has been revealed as a potential therapeutic target for HCC due to its involvement in the proliferation, invasion, and metastasis of HCC (21).

RFX6 is an important protein involved in beta-cell maturation and function in the pancreas (22). TCGA data showed its high expression in HCC and was negatively correlated with the prognosis of HCC patients. DTX2, which was predicted as a possible interactive protein of RFX6, is a regulator of Notch signaling (23). Our study demonstrated that a microRNA promoted the mechanism of HCC invasion and metastasis by regulating target genes and activating the Notch pathway, as well as affects the immune response in HCC. miRNA-542-3p has been reported as a potential microRNA inhibiting HCC clinical development, but its regulatory mechanism is not clear. Here, we found that miRNA-542-3p regulates RFX6, raising the expression of DTX2 resulting in the stable expression of Notch1 and activating the Notch pathway, which primarily affected the invasion and immune responses in HCC.

## Methods

### Human Cancer Cell Xenograft Model

All animal work was approved by the Institutional Animal Care and Use Committee (IACUC) of Southern Medical University. Liver cancer cells (3 × 10^6^) were implanted into the skeletal muscle of the hind limbs of 3~4-week-old BALB/c nude mice (nu/nu). One week after transplantation, the diameter of the tumors was measured every 3 days. Tumors were recovered and weighed after 3 weeks.

### Cell Culture

Human hepatocellular liver carcinoma cell lines (HepG2, Huh7) were purchased from the American Type Culture Collection (ATCC, Manassas, VA, USA). The normal liver cell LO2 was purchased from the American Type Culture Collection (ATCC, Manassas, VA, USA) and cultured in Roswell Park Memorial Institute 1640 medium (Gibco, USA) supplemented with 10% fetal bovine serum (FBS, HyClone, UT, USA) and 1% penicillin–streptomycin (Thermo). HepG2 and Huh7 were cultured in Dulbecco’s modified Eagle medium (DMEM, Gibco, USA) supplemented with 10% FBS (HyClone, UT, USA) and 1% penicillin–streptomycin (Pen/Strep) (Gibco) at 37°C with 5% CO_2_. For the co-culture experiment, 2 × 10^7^/ml T cells [stored in our laboratory (24)] were activated in an activated system stimulated by 3 μg/ml anti-CD3 antibody (BD Biosciences Cat# 555329, RRID: AB_395736) and 1 μg/ml anti-CD28 antibody (BD Biosciences Cat# 555725, RRID: AB_396068). Tumor cells (2 × 10^6^) were inoculated into 12-well plates and activated T cells were added into each well. The cells were mixed and co-cultured for 2 days.

### Establishment of the Transfected Cell Lines

The vectors expressing RFX6-specific siRNA (RiboBio, Cat# SI00701841, Cat# SI04193749, Cat# SI04249056) and DTX2-specific siRNA (RiboBio, Cat# SI04728740) were utilized in the study. Vectors expressing human RFX6 cDNA and DTX2 cDNA were transfected into cells. Cells were selected with puromycin (2 μg/ml, GeneChem) for 3 days for stable transfection. Details of the siRNA sequence are listed as follows:

RFX6 siRNA-1: TACGCTCATAATGATGTACAA, RFX6 siRNA-2: AAGCCGAGGAAGTGTCATTAA, RFX6 siRNA-3: CAGCGACGCTGTGAAGAATGA, DTX2 siRNA: GAGTGTTCTGATGTCAGCCATTGGA

### Western Blot Analysis

Total proteins were extracted from the cells by lysis buffer, and samples were separated on 8%–15% SDS-PAGE and transferred to nitrocellulose membranes, which were blocked with blocking buffer (5% skim milk in PBS with 0.05% Tween 20) and incubated with primary antibody in the blocking buffer. After being washed three times with the blocking buffer, the membrane was probed with secondary antibody and developed with SuperSignal West Pico (Thermo Fisher Scientific).

### Quantitative PCR Analysis

Real-time PCR analysis was performed using the StepOnePlus Real-Time PCR System (Applied Biosystems) with FastStart Universal SYBR Green Master (Roche) as previously reported (25). The primer sets used are as follows:

GAPDH-F: GAACGGGAAGCTCACTGG, GAPDH-R: GCCTGCTTCACCACCTTCT, RFX6-F: GGTACCATGGCCAAGGTCCCGGAGCTG, RFX6-R: TCTAGATTAAGTGCCTCCAGCTGCTGTTC, DTX2-F: GGTACCATGGCCATGGCCCCAAGCCC, DTX2-R: GGTACCTCACTGCTGCTCCAGGCAGTCC, U6-F: GCTTCGGCAGCACATATACTAAAAT, U6-R: CGCTTCATGAATTTGCGTGTCAT, miRNA-542-3p: TGTGACAGATTGATAACTG, miR-542-3p-RFX6 mut-F: TGATTTGACAGTGTTAGCAGCATTCCGATTCTATG, miR-542-3p-RFX6 mut-F-R: CTGCTAACACTGTCAAATCATCACACTAATGCAC

The PCR conditions were as follows: 10 min at 95°C, 40 cycles of 15 s at 95°C, and 1 min at 60°C. The average Ct value for each gene was determined from triplicate reactions and normalized with the amount of GAPDH for the gene and U6 for miRNA.

### Cell Proliferation, Apoptosis, Migration Assay, and Invasion Assay

Cells transfected with various plasmids were seeded onto 96-well plates (Corning Inc., Corning, NY, USA) at a density of 1 × 10^4^ cells/well in 96-well plates. At different time points (24, 48, and 72 h) after plating, the number of cells was assessed using the Cell Counting Kit 8 according to the protocols of the manufacturer (Dojindo, Tokyo, Japan). The transfected cell lines undergoing apoptosis were distinguished from live and necrotic cells by using Annexin-V and propidium iodide (PI) staining kit (Calbiochem, San Diego, CA, USA). All experiments were independently repeated three times.

For cell invasion assay, 1.5 × 10^5^ cells in serum-free medium were seeded into a Matrigel-coated chamber (8 μm pore size, Corning Incorporated, NY, USA), and the lower chamber was immediately filled with 500 μl of 1640 medium with 10% FBS as a chemoattractant. After 24 h of incubation, the non-invading cells were removed from the upper chamber by a cotton swab. The membranes were fixed with methanol and stained by 0.1% crystal violet. The data were represented as mean ± standard deviation (SD), *n* = 3.

### Dual-Luciferase Reporter Assay

Cells were seeded in triplicate onto 6-well plates at a density of 4 × 10^5^ cells/well for 48 h and transfected with 0.3 μg of certain plasmids separately together with 30 ng of pGMR TK Renilla plasmid (Genomeditech, Shanghai, China) using Lipofectamine™ 3000 reagent (Invitrogen, Carlsbad, USA). Luciferase and Renilla activities were measured using the Dual-Luciferase Reporter Assay Kit (Promega, Madison, USA) after 48 h of transfection.

### Quantitative Real-Time PCR for Chromatin Immunoprecipitation Analysis (CHIP-qPCR Assay)

Formaldehyde was directly added into the cell culture medium, after shaking and fixation for 10 min. Glycine was quickly added into the mixture for 5 min and then cells were washed with PBS and scraped off into ep tubes. Specific antibody, IgG, and magnetic beads (or protein A/G) that bind antibodies were added into ultrasonic centrifugal products as mentioned above. The ultrasonic products were divided into two parts, and the specific antibody and IgG were added and were rotated overnight. Magnetic beads were added and rotated for 4 h. The mixture was washed with dilute buffer solution, low salt solution, high salt solution, lithium chloride solution, and TE buffer solution seven times, and the products were put into the eluting buffer at 65°C overnight. The elution products were purified with DNA purification kit prepared for ChIP detection. Primers were designed according to the range of 2,000–3,000 bp upstream of the ORF region of the prediction gene in stages. Samples were screened by quantitative PCR with primers. The bound primers screened by PCR were verified by qPCR with input and IP samples. The sequence of the ChIP-qPCR is listed as follows: F: AATGAGTGAGCAGGCGAAGG and ChIP-R: GAAATGTAGTCCCGGTAGGGC.

### Co-Immunoprecipitation Assay

Cells were lysed in RIPA buffer containing protease and phosphatase inhibitors, and cells were collected after 12,000×*g* for 10 min at 4°C centrifugation for immunoprecipitation assays. Supernatants were immunoprecipitated with antibodies followed by incubation with magnetic protein A/G beads (Pierce) for 2 h at 4°C. The immune complexes were washed three times with PBS buffer, resuspended in SDS-PAGE buffer, and analyzed by Western blot analysis.

### Statistical Analysis

Data were analyzed using SPSS 20.0 and two-tailed independent Student’s *t*-test, and *p <*0.05 was considered significant. Two patient cohorts were compared by using the Kaplan–Meier survival plot and log-rank *p*-values were calculated.

## Results

### RFX6 Is Highly Expressed in Hepatocellular Carcinoma and Negatively Correlated With Prognosis

**Figure 1:**
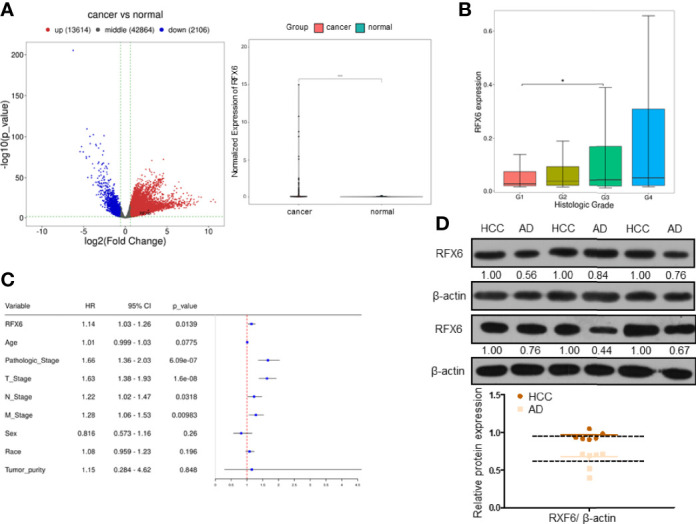
RFX6 is highly expressed in hepatocellular carcinoma and negatively correlated with prognosis. **(A)** TCGA data analysis of differentially expressed genes in liver hepatocellular carcinoma tissues (*n* = 374) compared with adjacent normal tissues (*n* = 50) (left panel). The paired comparison of RFX6 in the TCGA database, containing human breast cancers (*n* = 50) and tumor adjacent normal tissues (*n* = 50) (right panel, *p < 0.05). **(B)** Wilcoxon test was used to test the difference of RFX6 expression in different groups of clinical data to demonstrate the correlation between RFX6 expression and histologic grade. **(C)** Multivariate Cox regression analysis of patients based on the expression level of RFX6. **(D)** The protein levels of RFX6 were higher in liver cancer tissue samples than in adjacent normal tissues (six pairs).

The RNA-seq data of liver hepatocellular carcinoma (LIHC) and paired adjacent normal tissues from the TCGA database (https://portal.gdc.cancer.gov/) showed RFX6 was overexpressed in hepatocellular carcinoma (**Figure 1A**). Positive RFX6 expression level was closely correlated with histologic grade (**Figure 1B**), pathologic stage, and TNM stage (**Figure 1C**). The relative protein expression of RFX6 in hepatocellular carcinoma tissues was higher than that in adjacent normal tissues (**Figure 1D**). In the meantime, we examined the expression levels of RFX6 mRNA and protein in HepG2, Huh7, and LO2 cell lines. As shown in **Supplementary Figures 1A, B**, the expression levels of RFX6 mRNA and protein were increased in HepG2 and Huh7 when compared with the normal liver cell LO2.

**Figure 2:**
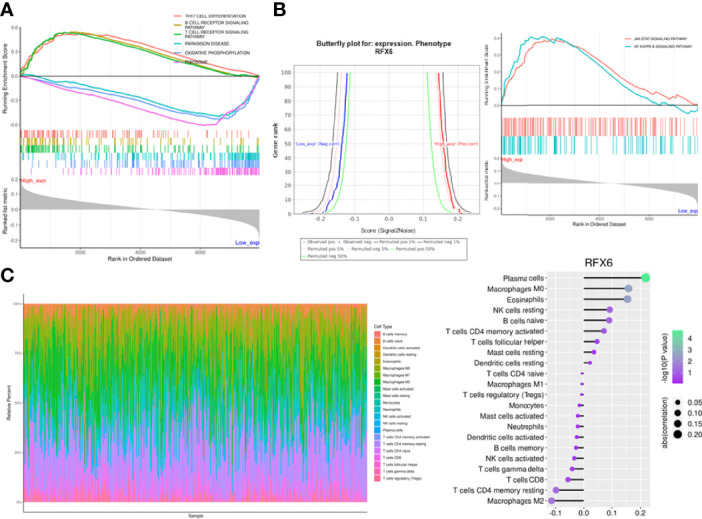
RFX6 affects immune cell infiltration in the hepatocellular carcinoma microenvironment. **(A)** Gene set enrichment analysis of the KEGG pathways related with different RFX6 expression levels. **(B)** Butterfly plot for RFX6 expression phenotype (left panel). EMT pathway enrichment according to different RFX6 expression levels (right panel). **(C)** CIBERSORT bar plot of different immune cell infiltration in the hepatocellular carcinoma microenvironment (left panel). Point plot of immune cell correlation with RFX6 (right panel).

According to RFX6 expression value, the samples were divided into high and low expression groups (median division), and the difference between the two groups was calculated. GSEA (gene set enrichment analysis) revealed RFX6 may be positively related to TH17 cell differentiation and B-cell and T-cell receptor signaling pathway (**Figure 2A**). KEGG analysis indicated that RFX6 was possibly involved in multiple pathways. It is worth noting that the high expression of RFX6 was positively correlated with EMT pathways in cancer, namely, the NF-kB and JAK–STAT pathway (**Figure 2B**). Due to the possible role of RFX6 in tumor immune response, we estimated the immune infiltration with CIBERSORT (26). The correlation between RFX6 expression and immune infiltration of each immune cell type was shown in the bubble diagram. It is worth noting that a weak significant correlation could be presumed between the expression of RFX6 and CD8 T cells (*R* = −0.056, *p* = 0.28), and the details are shown in **Figure 2C**.

### Expression of RFX6 Promotes Tumorigenesis and T-Cell Immune Response of Hepatocellular Carcinoma

**Figure 3:**
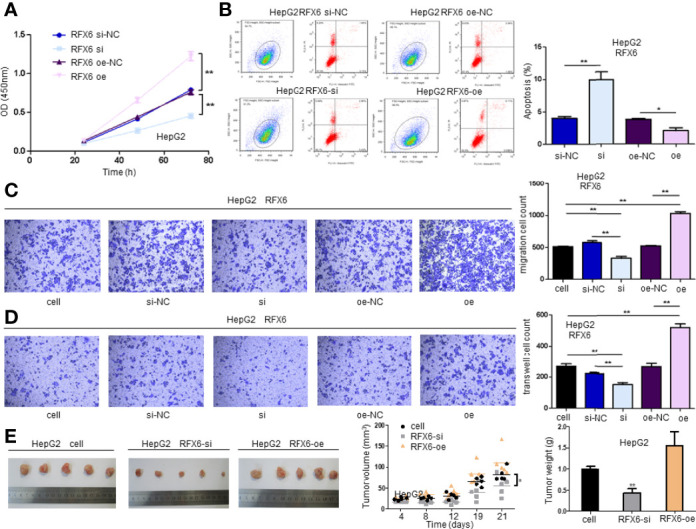
Expression of RFX6 promotes the tumorigenesis of hepatocellular carcinoma. **(A)** The proliferation of HepG2 cells when altered by the expression of RFX6 in cells. The cell number was determined using the CCK-8 assay. Data were represented as mean ± standard deviation (SD), *n* = 3, **p < 0.01. **(B)** The apoptosis of HepG2 before and after RFX6 alteration. Upper right (UR, PI^+^Annexin^+^) and lower right (LR, PI^−^Annexin^+^) were counted as apoptotic cells. Data were represented as mean ± standard deviation (SD), *p < 0.05, **p < 0.01. **(C)** Expression of RFX6 promoted the migration of HepG2 cells using a Transwell assay. Data were represented as mean ± standard deviation (SD), *n* = 3, **p < 0.01. **(D)** The knockdown of RFX6 could significantly inhibit the invasion of HepG2 cells. Data were represented as mean ± standard deviation (SD), *n* = 3, **p < 0.01. **(E)** The knockdown of RFX6 suppressed the tumor growth of HepG2 cells in nude mice,n=5, *p < 0.05, **p < 0.01.

The high expression of RFX6 in HCC and its negative role in the prognosis and CD8^+^ T-cell infiltration support the notion that RFX6 helps promote liver hepatocellular tumorigenesis and may be related to antitumor immunity mediated by CD8^+^ T cells. In order to investigate the roles of RFX6 in HCC, we regulated the expression level of RFX6 in HepG2 and Huh7 by knockdown and overexpression (**Supplementary Figures 1C, D**). Downregulating RFX6 decreased the cellular proliferation and survival of HepG2 and Huh7, while increasing the expression of RFX6 in cells could stimulate the proliferation and inhibit cell apoptosis (**Figures 3A, B** and **Supplementary Figures 2A, B**). Transwell assays indicating the expression of RFX6 significantly promoted the migration and invasion abilities of HepG2 and Huh7 (**Figures 3C, D** and **Supplementary Figures 2C, D**). Knockdown of RFX6, by contrast, got the opposite results of suppression of tumor cell migration and invasion (**Figures 3C, D** and **Supplementary Figures 2C, D**). Furthermore, the suppression of RFX6 expression in HepG2 cells significantly reduced the growth of xenograft formed by HepG2 cells in immunodeficient mice (**Figure 3E**).

**Figure 4:**
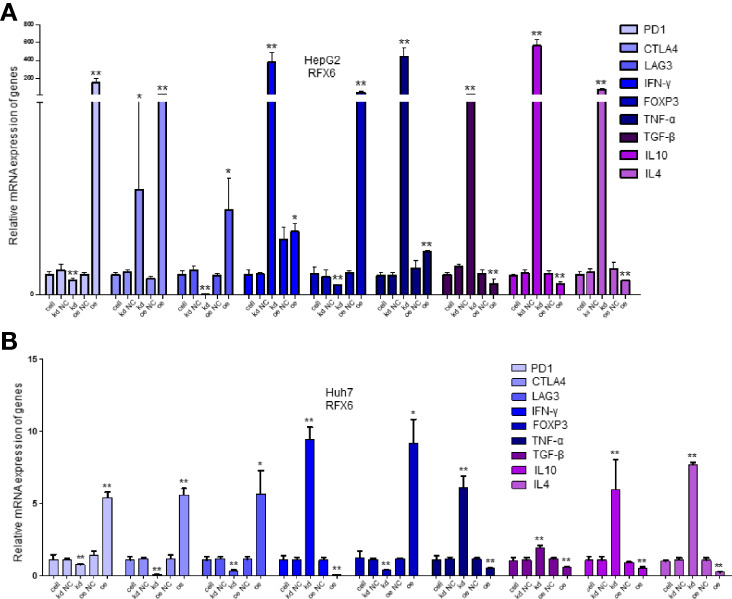
Significantly expressed marker genes of immune cells under different RFX6 expression levels. **(A)** mRNA expression changes of immune cell receptor marker genes in HepG2. Data were represented as mean ± standard deviation (SD), *n* = 3, *p < 0.05, **p < 0.01. **(B)** Relative expression of immune cell receptor mRNA in Huh7 after RFX6 alteration. Data were represented as mean ± standard deviation (SD), *n* = 3, *p < 0.05, **p < 0.01.

As the results in **Figures 2C, D** show, RFX6 may be relevant with the activation of T cells in HCC. Therefore, we detected 23 known marker genes of different immune cell types by qPCR in the mixed cells co-cultured with T cells after changing the expression amount of RFX6 in HepG2 and Huh7. It can be noted that positive RFX6 expression was relevant with regulatory T cells and inversely correlated with the dysfunction of progressive T cell by affecting the receptors of effector T cells such as CTLA-4, PD-1, and LAG3 (**Figures 4A, B** **)**. The list of 23 markers is shown in **Table 1**. These results indicate that RFX6 expression induced progressive T-cell exhaustion.

**Table 1:** The sequences of 23 marker genes regarding tumor immunity.

Primer name	5′ sequence 3′
Has-TRIB3-F	CTAGGACCACCCTACTACAC
Has-TRIB3-R	CACCTGATAAGCACCCAAGC
Has-CTLA4-F	GACAGAGCTGGGATGTTTCTG
Has-CTLA4-R	CGGCTATAAACGTCTCATACG
Has-PD1-F	GTGAGCATGAAACTATGCACC
Has-PD1-R	GCCACTTAAGGAACCAGTGC
Has-LAG3-F	GCTTCAACGTCTCCATCATG
Has-LAG3-R	GGCTCACATCCTCTAGTCG
Has-TIM3-F	GGCATCTACATCGGAGCAG
Has-TIM3-R	GTGGTTGGATCTATGGCATTG
Has-IFNγ-F	CGGTAACTGACTTGAATGTCC
Has-IFNγ-R	CAGGCAGGACAACCATTAC
Has-IL2-F	CTCACAGTAACCTCAACTCC
Has-IL2-R	CCTCCAGAGGTTTGAGTTC
Has-FOXP3-F	GAAGGTCTTCGAAGAGCCAG
Has-FOXP3-R	GTCGGATGATGCCACAGATG
Has-TNFα-F	GGTATGAGCCCATCTATCTGG
Has-TNFα-R	CAGAAGAGGTTGAGGGTGTC
Has-TGFβ-F	GCAAGACTATCGACATGGAG
Has-TGFβ-R	GGTTTCCACCATTAGCACG
Has-IL6-F	GTAGTGAGGAACAAGCCAGAG
Has-IL6-R	GCAGGAACTCCTTAAAGCTG
Has-IL10-F	GAACCAAGACCCAGACATCAAG
Has-IL10-R	CACCCTGATGTCTCAGTTTCG
Has-IL17-F	CTGATGGGAACGTGGACTAC
Has-IL17-R	GCCAAGTGTTACCTCTGAAGC
Has-IL4-F	CAGTTCCACAGGCACAAGC
Has-IL4-F	CGTACTCTGGTTGGCTTCC
Has-CD3-F	CTTCACACACAGACTGTTGTC
Has-CD3-R	CTAGCATCTGCGCTTTCTC
Has-CD80-F	CTTCAGAGACTATCTGATTTCC
Has-CD80-R	GACTACTGCTTTGACGTACC
Has-CD86-F	CAAGCCATAGTGGAGAGAAC
Has-CD86-R	CTGCTGTCTGTCTTATGTCC
Has-CD68-F	CCATCTTGCTGCCTCTCATC
Has-CD68-R	GTCTTTGAGCCAGTTGCGTG
Has-CD206-F	GTAATGCATTTGCGTGGCTG
Has-CD206-R	GCAATGTGCTGTCTTCCAG
Has-CD163-F	GACTCTTGGGACTTGGACG
Has-CD163-R	CCACAAGGAAGACTCATTCC
Has-LEF1-F	CTAATGCACGTGAAGCCTCAGC
Has-LEF1-R	GTCTCTTGCAGACCAGCCTG
Has-TCF7-F	GATCTCATGGAAACTGGCCAG
Has-TCF7-R	GCTGGCAAGACAGATGGTAC
Has-GZMB-F	GCTTCCTGATACGAGACGAC
Has-GZMB-R	CGATCTTCCTGCACTGTCATC

### miRNA-542-3p Negatively Regulates RFX6 and Positively Correlates With the Prognosis of Human Hepatocellular Carcinoma

**Figure 5:**
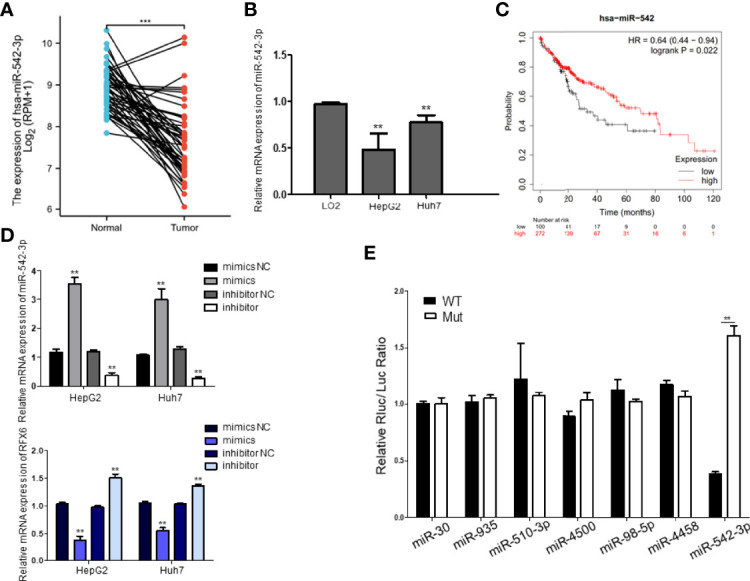
miRNA-542-3p negatively regulates RFX6 and positively correlates with the prognosis of human hepatocellular carcinoma. **(A)** The comparison of the expression level of miR-542-3p in human hepatocellular carcinoma (*n* = 374) and tumor adjacent normal tissues (*n* = 50) in the TCGA database, ***p < 0.001. **(B)** The expression of miR-542-3p was significantly reduced in liver cancer cells. Data were represented as mean ± standard deviation (SD), *n* = 3, **p < 0.01. **(C)** Log-rank (Mantel–Cox) survival test of hepatocellular carcinoma patients based on the levels of miR-542 (low expression *n* = 100, high expression *n* = 272), and *p*-values were indicated. **(D)** Alteration of miR-542-3p expression inversely correlated with the levels of RFX6 mRNA. Data were represented as mean ± standard deviation (SD), *n* = 3, **p < 0.01. **(E)** miR-542-3p targeted with the predicted sites within the 3′UTR of RFX6 and mutation of the predicted target sites rescued the inhibitory effects of RFX6. Data were represented as mean ± standard deviation (SD), *n* = 3, **p < 0.01.

Considering the importance of RFX6 in promoting liver cell tumorigenesis and the significant role of miRNA in tumor suppression and oncogenesis, we used the miRDB prediction website (http://mirdb.org/) to predict miRNAs that might target RFX6. According to the expression level in liver hepatocellular carcinoma and target score, 36 miRNAs (list shown in **Table 2**) were selected as potential regulators of RFX6 mRNA. Through analysis of the TCGA datasets, we found only one candidate—miRNA-542-3p—that was highly expressed in tumor adjacent normal tissues, compared with liver hepatocellular carcinoma tissues (**Figure 5A**). A consistent result was found in the hepatocellular carcinoma cell lines HepG2 and Huh7 (**Figure 5B**). Moreover, the expression level of miRNA-542-3p was negatively related with the prognosis of HCC patients (**Figure 5C**). To determine the impact of miRNA-542-3p on the expression of RFX6, we used miRNA mimics and inhibitor to demonstrate that the expression levels of miRNA-542-3p were inversely correlated with the expression level of RFX6 (**Figure 5D**). Dual-luciferase reporter assay detected that mutating the predicted sites of miRNA-542-3p in the 3′UTR of RFX6 mRNA significantly heightened the fluorescence ratio (**Figure 5E**). These data confirmed that miRNA-542-3p regulated RFX6 in hepatocellular carcinoma cells.

**Table 2:** miRNA prediction results of RFX6 mRNA.

Target rank	Target score	miRNA name	Gene symbol	Gene description
1	99	hsa-miR-30b-5p	RFX6	Regulatory factor X6
2	99	hsa-miR-30a-5p	RFX6	Regulatory factor X6
3	99	hsa-miR-30c-5p	RFX6	Regulatory factor X6
4	99	hsa-miR-30d-5p	RFX6	Regulatory factor X6
5	99	hsa-miR-30e-5p	RFX6	Regulatory factor X6
6	97	hsa-miR-935	RFX6	Regulatory factor X6
7	94	hsa-miR-376c-3p	RFX6	Regulatory factor X6
8	91	hsa-miR-510-3p	RFX6	Regulatory factor X6
9	88	hsa-miR-664a-3p	RFX6	Regulatory factor X6
10	85	hsa-miR-4500	RFX6	Regulatory factor X6
11	82	hsa-let-7d-5p	RFX6	Regulatory factor X6
12	82	hsa-let-7c-5p	RFX6	Regulatory factor X6
13	82	hsa-let-7i-5p	RFX6	Regulatory factor X6
14	82	hsa-miR-98-5p	RFX6	Regulatory factor X6
15	82	hsa-let-7a-5p	RFX6	Regulatory factor X6
16	82	hsa-let-7b-5p	RFX6	Regulatory factor X6
17	82	hsa-let-7g-5p	RFX6	Regulatory factor X6
18	82	hsa-let-7e-5p	RFX6	Regulatory factor X6
19	82	hsa-miR-4458	RFX6	Regulatory factor X6
20	82	hsa-let-7f-5p	RFX6	Regulatory factor X6
21	81	hsa-miR-548an	RFX6	Regulatory factor X6
22	81	hsa-miR-153-5p	RFX6	Regulatory factor X6
23	77	hsa-miR-4501	RFX6	Regulatory factor X6
24	77	hsa-miR-648	RFX6	Regulatory factor X6
25	76	hsa-miR-587	RFX6	Regulatory factor X6
26	76	hsa-miR-3194-3p	RFX6	Regulatory factor X6
27	75	hsa-miR-542-3p	RFX6	Regulatory factor X6
28	75	hsa-miR-9-5p	RFX6	Regulatory factor X6
29	75	hsa-miR-548m	RFX6	Regulatory factor X6
30	75	hsa-miR-6514-5p	RFX6	Regulatory factor X6
31	75	hsa-miR-653-3p	RFX6	Regulatory factor X6
32	75	hsa-miR-10523-5p	RFX6	Regulatory factor X6
33	72	hsa-miR-4699-3p	RFX6	Regulatory factor X6
34	71	hsa-miR-506-3p	RFX6	Regulatory factor X6
35	71	hsa-miR-124-3p	RFX6	Regulatory factor X6
36	70	hsa-miR-203a-3p	RFX6	Regulatory factor X6

### Downregulation of RFX6 by miRNA-542-3p Affects the Invasion and Oncogenesis of Hepatocellular Carcinoma Cells

**Figure 6:**
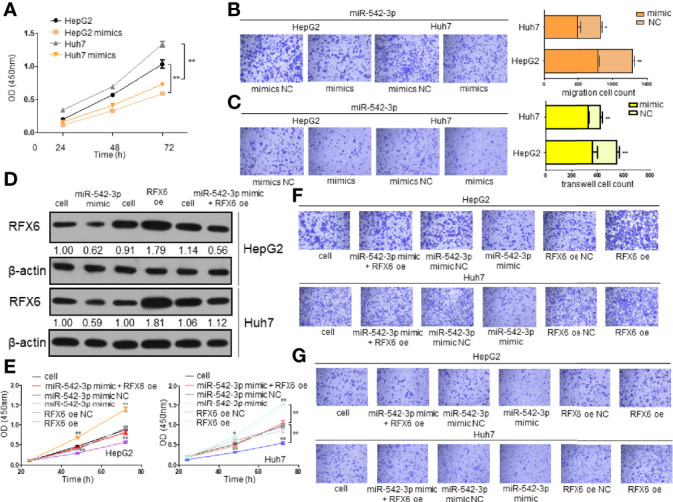
Downregulation of RFX6 by miRNA-542-3p affects the invasion and oncogenesis of hepatocellular carcinoma cells. **(A)** Overexpression of miR-542-3p reduced the proliferation of liver cancer cells. The cell number was determined using the CCK-8 assay. Data were represented as mean ± standard deviation (SD), *n* = 3, **p < 0.01. **(B)** miR-542-3p overexpression could significantly suppress the migration of HepG2 and Huh7 cells. Data were represented as mean ± standard deviation (SD), *n* = 3, *p < 0.05, **p < 0.01. **(C)** Overexpression of miR-542-3p reduced the invasion ability of liver cancer cells. Data were represented as mean ± standard deviation (SD), *n* = 3, **p < 0.01. **(D)** Overexpression of RFX6 could reverse the protein level of RFX6 inhibited by miR-542-3p. **(E)** Proliferation of liver cancer cells when rescuing RFX6 expression. The cell number was determined using the CCK-8 assay. Data were represented as mean ± standard deviation (SD), *n* = 3, *p < 0.05, **p < 0.01. **(F)** Migration ability of liver cancer cells before and after rescuing the expression of RFX6. **(G)** Rescuing the expression of RFX6 affected the invasion ability of HepG2 and Huh7 cells.

Since miRNA-542-3p suppresses RFX6 expression in hepatocellular carcinoma cells, we wonder if such regulation affects the tumorigenesis of liver cells. To investigate the role of miRNA-542-3p, we induced miRNA-542-3p expression by mimics in HepG2 and Huh7 cells. The proliferation, migration, and invasion of HCC cells were significantly inhibited by the overexpression of miRNA-542-3p (**Figures 6A–C**). Moreover, a variety of tumor immune-related cytokines such as IFN-γ, TNF-α, TGF-β, IL10, and IL4 were notably increased in cells after upregulating the expression of miRNA-542-3p in HepG2 and Huh7 cells and co-cultured with T cells (**Supplementary Figure 3A**). However, due to the different effects of IFN-γ, TNF-α, TGF-β, IL10, and IL4 on T-cell function, the significance of such change was limited. Consistently, overexpression of miRNA-542-3p could suppress the tumorigenesis induced by RFX6 overexpression (**Figures 6D–G** and **Supplementary Figures 3B, C**). Therefore, these results indicate that miRNA-542-3p could inhibit HCC tumorigenesis by suppressing the expression of RFX6.

### RFX6 Contributes to the Invasion Through The Notch Pathway by Promoting the Translation of DTX2 in Hepatocellular Carcinoma

**Figure 7:**
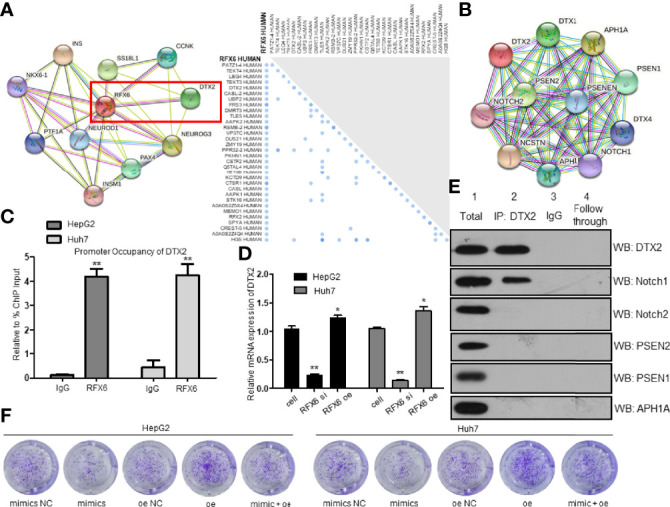
Expression of RFX6 promotes the translation of DTX2 and participates in the Notch pathway. **(A)** STRING analysis predicted a network of proteins that interacted with RFX6 (left panel). UniProt analysis of probable interacting proteins of RFX6 (right panel). **(B)** STRING analysis of possible interacting proteins of DTX2. **(C)** Promoter occupancy of the *DTX2* gene by RFX6 performed by ChIP-qPCR. Data were represented as mean ± standard deviation (SD), *n* = 3, **p < 0.01. **(D)** Expression of RFX6 increased the mRNA expression of DTX2. Data were represented as mean ± standard deviation (SD), *n* = 3, *p < 0.05, **p < 0.01. **(E)** Notch1 was confirmed to interact with DTX2 in HepG2 cells by co-IP assay. **(F)** Proliferation of liver cancer cells was retrained by the downregulation of DTX2 conducted by miR-542-3p.

As a transcription factor, we predicted that RFX6 probably functioned by regulating the stability of mRNAs related to tumorigenesis in HCC. For the purpose of understanding the mechanism of RFX6 promoting HCC tumorigenesis, we used a functional association network database STRING (https://string-db.org/) and UniProt (https://www.uniprot.org/) to predict the targets that might interact with RFX6. The intersection of predicted outcomes was DTX2, a probable E3 ubiquitin-protein ligase (**Figure 7A**). DTX2 regulates the Notch pathway *via* ubiquitin ligase activity *in vitro*.

Most of the potential targets were involved in the Notch pathway (**Figure 7B**). As RFX6 was reported as a transcription factor, we used ChIP-qPCR to determine the target gene of RFX6. So, we confirmed the interaction between RFX6 and DTX2 (**Figure 7C**). Meanwhile, the mRNA level of DTX2 was positively correlated with the protein levels of RFX6 (**Figure 7D** and **Supplementary Figure 3D**). These data support the notion that RFX6 helps regulate the transcription of DTX2. To determine the interacting protein of DTX2 in HCC, we used co-IP assay to identify Rfx6 (**Supplementary Figure 3E**) and STRING candidates of the Notch pathway. Of all the six potential proteins, Notch1 was confirmed to interact with DTX2 (**Figure 7E**). Furthermore, we used the miR-542-3p mimic to suppress the expression of RFX6, and the mRNA expression level of DTX2 was further inhibited (**Supplementary Figure 3F**). The HCC tumorigenesis level was retrained by the downregulation of DTX2 conducted by miR-542-3p (**Figure 7F** and **Supplementary Figure 3G**).

## Discussion

Hepatocellular carcinoma develops predominantly with cirrhosis and hepatitis. The absence of curative treatment methods and rapid drug resistance result in high mortality rate. It is urgent to conduct an extensive research toward the mechanism of HCC development and to decrease cancer cell resistance to treatment and immune response. RFX6 is known as a transcription factor required for endocrine pancreas development and regulates beta-cell maturation and function. Our study discovered that RFX6 was overexpressed in HCC and its expression predicts poor prognosis of HCC patients, but the role of RFX6 in tumorigenesis remains unclear. Through knockdown of RFX6 in HCC cells, we found that the absence of RFX6 induced a higher cell apoptosis rate and poor proliferation, migration, and invasion ability. DTX2 is a regulator of the Notch signaling pathway involved in cell fate and tumor immunology. We discovered that RFX6 could promote the transcription of DTX2 and was regulated by miRNA-542-3p in HCC, which positively affected the tumorigenesis and was involved in T-cell function in HCC.

Considering the important roles of RFX6 in tumorigenesis, we investigated the critical regulator of RFX6 by the dual-luciferase reporter assay system. Published data and our own data indicate that miRNA-542-3p is downregulated in different types of human cancers, including breast cancer, hepatocellular carcinoma, and lung cancer. These findings suggest that miRNA-542-3p is involved in tumor suppression. We found that miRNA-542-3p is positively correlated with good prognosis in HCC. There is limited information of the regulating mechanism of miRNA-542-3p. Therefore, identifying the upstream suppressive pathways that regulate the expression of miRNA-542-3p is a promising research area. Our discovery of the functional link between miRNA-542-3p, RFX6, and DTX2 plays critical roles in hepatocellular carcinoma and provides potential therapeutic targets for hepatocellular carcinoma treatment.

## Conclusions

We discovered that the miRNA-542-3p–RFX6–DTX2–NOTCH1 regulatory pathway plays significant roles in tumor progression and provides new potential therapeutic targets for live hepatocellular carcinoma.

## Data Availability Statement

The datasets presented in this study can be found in online repositories. The names of the repository/repositories and accession number(s) can be found in the article/**Supplementary Material**.

## Ethics Statement

The studies involving human participants were reviewed and approved by the Medical Ethics Committee of the Second Affiliated Hospital of Xinjiang Medical University. The patients/participants provided their written informed consent to participate in this study. The animal study was reviewed and approved by the Experimental Animal Ethics Committee of the Second Affiliated Hospital of Xinjiang Medical University.

## Author Contributions

MS performed the cell function analysis of different cell lines and Western blot analysis and was a major contributor in writing the manuscript. MK carried out mechanistic research regarding co-IP and ChIP-qPCR, as well as interpreted the prognosis of HCC patients and carried out an *in vitro* tumorigenesis experiment. LZ was responsible for preprocessing the clinical tissue regarding cancer and adjacent normal tissue and conducted all qPCR analyses in this study. XP established the transfected cell lines and analyzed the miRNA candidate of the target gene. YX confirmed that the data/figure presentation accurately reflected the original and oversaw the fellows who participated in this study. All authors contributed to the article and approved the submitted version.

## Funding

This study was supported by the Natural Science Foundation of Xinjiang Uygur Autonomous Region (Grant No. 2017D01C249).

## Conflict of Interest

The authors declare that the research was conducted in the absence of any commercial or financial relationships that could be construed as a potential conflict of interest.

## Publisher’s Note

All claims expressed in this article are solely those of the authors and do not necessarily represent those of their affiliated organizations, or those of the publisher, the editors and the reviewers. Any product that may be evaluated in this article, or claim that may be made by its manufacturer, is not guaranteed or endorsed by the publisher.

## Supplementary Material

Supplementary Figure 1 Regulation of the mRNA and protein level of RFX6 in different cell lines. **(A)** RFX6 mRNA expression in HepG2, Huh7 and LO2 cells, *p <0.05, **p <0.01. **(B)** The protein levels of RFX6 in HepG2, Huh7 and LO2 cells. **(C)** Knockdown of RFX6 in HepG2, Huh7 and LO2 cells with three siRNAs, *p <0.05, **p <0.01. **(D)** The overexpression (oe) of RFX6 in HepG2, Huh7 and LO2 cells, **p <0.01.

Supplementary Figure 2 Expression of RFX6 promotes tumor progression of Huh7. **(A)** The proliferation of Huh7 cells altered by the expression of RFX6 in cells. The cell number was determined with CCK-8 assay. Data were represented as mean ± standard deviation (s.d.), n=3, *p <0.05, **p <0.01. **(B**) The apoptosis of Huh7 cells before and after RFX6 alteration. Upper right (UR, PI+Annexin+) and Lower right (LR, PI-Annexin+) were counted as apoptotic cells. Data were represented as mean ± standard deviation (s.d.), **p <0.01. **(C)** Expression of RFX6 promoted the migration of Huh7 cells using transwell assay. Data were represented as mean ± standard deviation (s.d.), n=3, **p <0.01. **(D)** The alteration of RFX6 expression could significantly regulated the invasion of Huh7 cells. Data were represented as mean ± standard deviation (s.d.), n=3, **p <0.01.

Supplementary Figure 3 miR-542-3p overexpression partially reversed the tumorigenesis induced by RFX6 overexpression. **(A)** mRNA expression changes of immune cell receptor marker genes in HepG2. Data were represented as mean ± standard deviation (s.d.), n=3, * p <0.05, **p <0.01. **(B)** Migration cell count of liver cancer cells. Data were represented as mean ± standard deviation (s.d.), n=3, *p <0.05, **p <0.01. **(C)** Invasion cell count by transwell assay of liver cancer cells. Data were represented as mean ± standard deviation (s.d.), n=3, **p <0.01. **(D)** The protein levels of RFX6 in HepG2 and Huh7 cells. **(E)** Rfx6 was confirmed to interact with DTX2 in HepG2 cells by Co-IP assay. **(F)** The expression of RFX6 protein (left panel) and mRNA level of DTX2 (right panel) after increasing the expression of miR-542-3p in HepG2 and Huh7 cells. **(G)** Colony numbers of HepG2 and Huh7 cells. Data were represented as mean ± standard deviation (s.d.), n=3, **p <0.01.

## Abbreviations

RFX6, regulatory factor X6; DTX2, deltex E3 ubiquitin ligase 2; KCTD9, potassium channel tetramerization domain containing 9; TRIP6, thyroid hormone receptor interactor 6; NOTCH1, Neurogenic locus notch homolog protein 1; ROC, receiver operator characteristic; GSEA, gene set enrichment analysis; KEGG, Kyoto Encyclopedia of Genes and Genomes; EMT, epithelial–mesenchymal transition; miRDB, MicroRNA Target Prediction Database; ChIP, chromatin immunoprecipitation assay.
