# Supplementary figures and images for: Inhibition of RFX6 Suppresses the Invasive Ability of Tumor Cells Through the Notch Pathway and Affects Tumor Immunity in Hepatocellular Carcinoma
Source: Front Oncol. 2021 Dec 20;11:801222. doi: 10.3389/fonc.2021.801222 (PMC8721116; doi:10.3389/fonc.2021.801222)

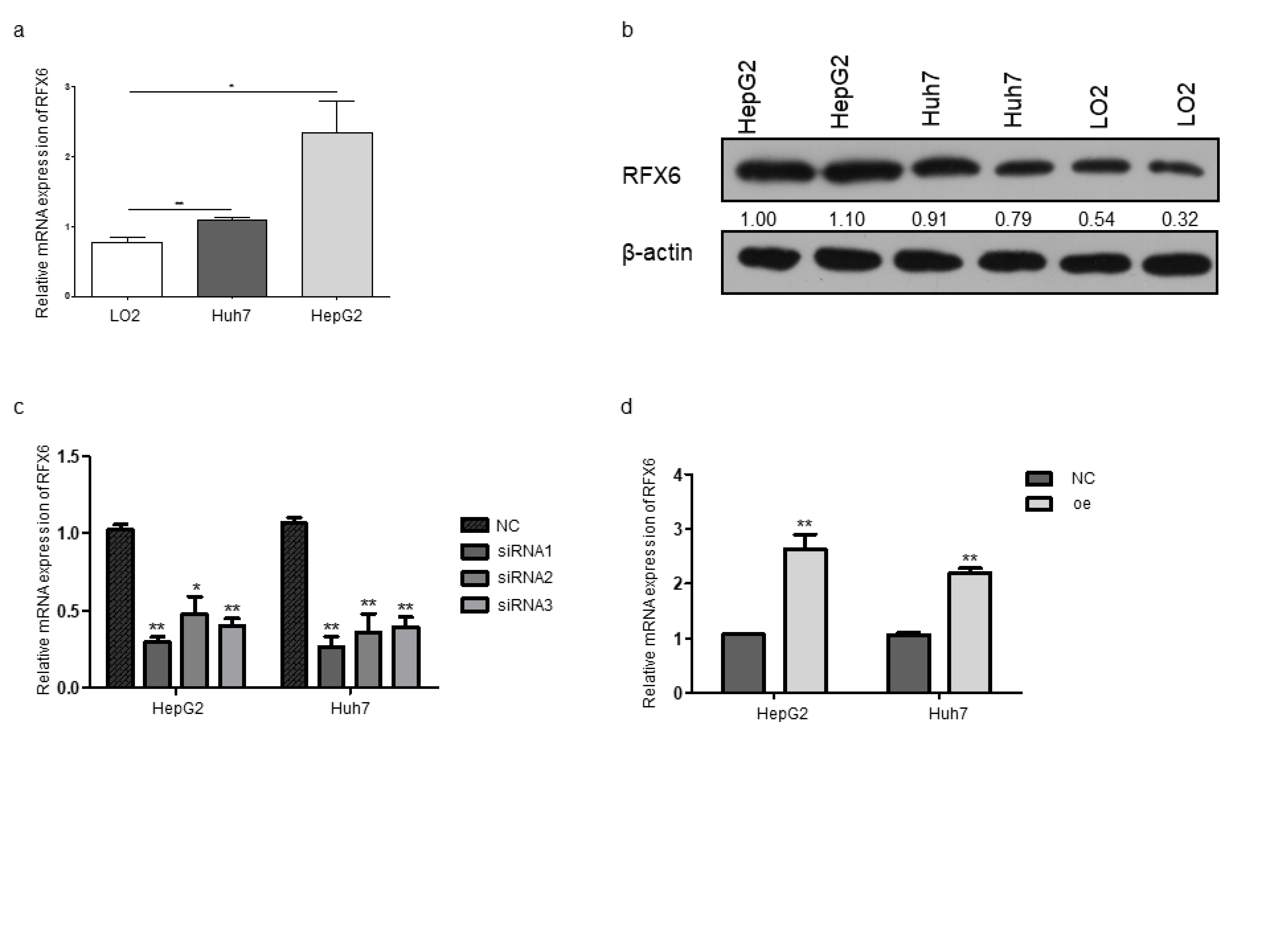

Supplement: Supplementary Figure 1 — Regulation of the mRNA and protein level of RFX6 in different cell lines. (A) RFX6 mRNA expression in HepG2, Huh7 and LO2 cells, *p <0.05, **p <0.01. (B) The protein levels of RFX6 in HepG2, Huh7 and LO2 cells. (C) Knockdown of RFX6 in HepG2, Huh7 and LO2 cells with three siRNAs, *p <0.05, **p <0.01. (D) The overexpression (oe) of RFX6 in HepG2, Huh7 and LO2 cells, **p <0.01. [file Image_1.tif]

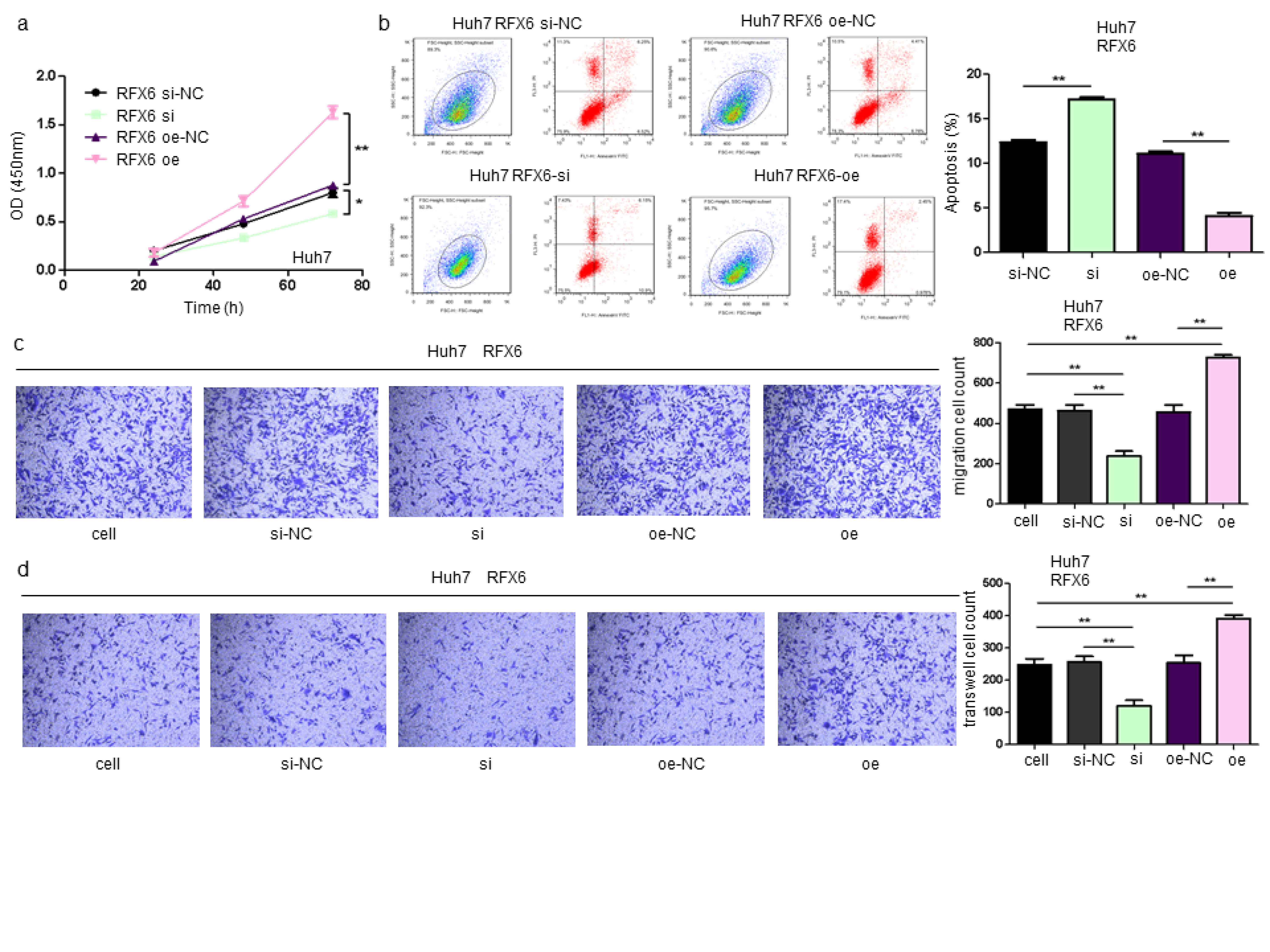

Supplement: Supplementary Figure 2 — Expression of RFX6 promotes tumor progression of Huh7. (A) The proliferation of Huh7 cells altered by the expression of RFX6 in cells. The cell number was determined with CCK-8 assay. Data were represented as mean ± standard deviation (s.d.), n=3, *p <0.05, **p <0.01. (B) The apoptosis of Huh7 cells before and after RFX6 alteration. Upper right (UR, PI+Annexin+) and Lower right (LR, PI-Annexin+) were counted as apoptotic cells. Data were represented as mean ± standard deviation (s.d.), **p <0.01. (C) Expression of RFX6 promoted the migration of Huh7 cells using transwell assay. Data were represented as mean ± standard deviation (s.d.), n=3, **p <0.01. (D) The alteration of RFX6 expression could significantly regulated the invasion of Huh7 cells. Data were represented as mean ± standard deviation (s.d.), n=3, **p <0.01. [file Image_2.tif]

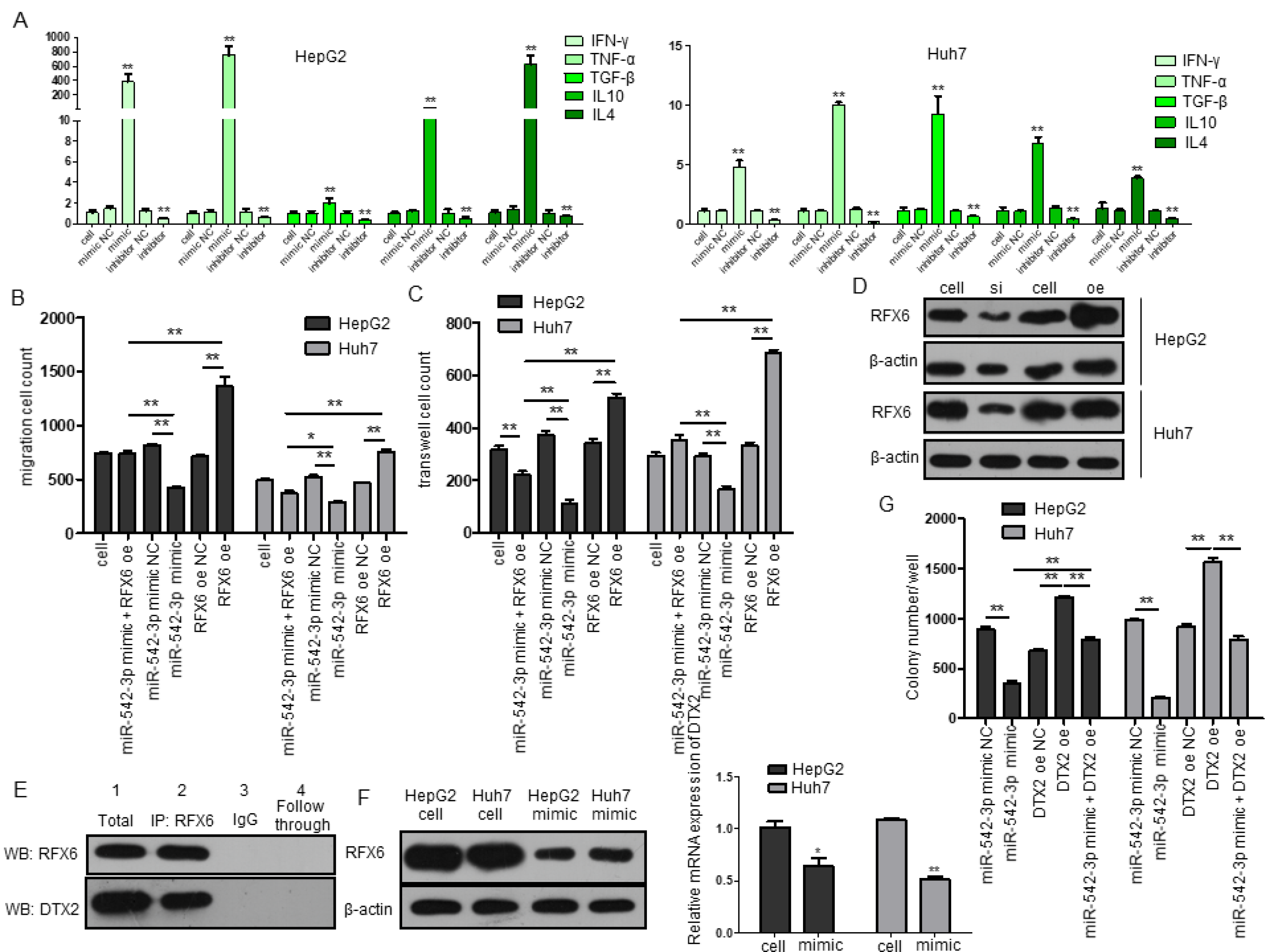

Supplement: Supplementary Figure 3 — miR-542-3p overexpression partially reversed the tumorigenesis induced by RFX6 overexpression. (A) mRNA expression changes of immune cell receptor marker genes in HepG2. Data were represented as mean ± standard deviation (s.d.), n=3, * p <0.05, **p <0.01. (B) Migration cell count of liver cancer cells. Data were represented as mean ± standard deviation (s.d.), n=3, *p <0.05, **p <0.01. (C) Invasion cell count by transwell assay of liver cancer cells. Data were represented as mean ± standard deviation (s.d.), n=3, **p <0.01. (D) The protein levels of RFX6 in HepG2 and Huh7 cells. (E) Rfx6 was confirmed to interact with DTX2 in HepG2 cells by Co-IP assay. (F) The expression of RFX6 protein (left panel) and mRNA level of DTX2 (right panel) after increasing the expression of miR-542-3p in HepG2 and Huh7 cells. (G) Colony numbers of HepG2 and Huh7 cells. Data were represented as mean ± standard deviation (s.d.), n=3, **p <0.01. [file Image_3.tif]
